# Predicting synthetic lethal genetic interactions in *Saccharomyces cerevisiae *using short polypeptide clusters

**DOI:** 10.1186/1477-5956-10-S1-S4

**Published:** 2012-06-21

**Authors:** Yuehua Zhang, Bo Li, Pradip K Srimani, Xuewen Chen, Feng Luo

**Affiliations:** 1 School of Computing, Clemson University, Clemson, SC 29634, USA; 2 Electrical Engineering and Computer Science Department, University of Kansas, Lawrence, KS 66045-7621, USA

## Abstract

**Background:**

Protein synthetic lethal genetic interactions are useful to define functional relationships between proteins and pathways. However, the molecular mechanism of synthetic lethal genetic interactions remains unclear.

**Results:**

In this study we used the clusters of short polypeptide sequences, which are typically shorter than the classically defined protein domains, to characterize the functionalities of proteins. We developed a framework to identify significant short polypeptide clusters from yeast protein sequences, and then used these short polypeptide clusters as features to predict yeast synthetic lethal genetic interactions. The short polypeptide clusters based approach provides much higher coverage for predicting yeast synthetic lethal genetic interactions. Evaluation using experimental data sets showed that the short polypeptide clusters based approach is superior to the previous protein domain based one.

**Conclusion:**

We were able to achieve higher performance in yeast synthetic lethal genetic interactions prediction using short polypeptide clusters as features. Our study suggests that the short polypeptide cluster may help better understand the functionalities of proteins.

## Background

Defining the functional relationships between proteins is essential to understand many aspects of biology. A classical approach of understanding gene functional relationships is to produce phenotype of combination mutant in two genes [1]; such relationships are called genetic interactions. Recently, high throughput methods [2-4] have been developed to generate large scale genetic interactions in model organisms, such as yeast [5], *Schizosaccharomyces pombe *[6] and *E. coli*. [7]. The large scale genetic interactions have attracted much attention as they are capable of defining the genome-wide functional relationships among proteins and are fundamental to comprehensive understanding of the organization of biological systems [5,8,9]. However, even with high throughput methods [2-4], experimental mapping of genetic interactions is still extremely labor intensive and one cannot screen genome-wide combinations in multiple cell organisms with ten thousands of genes as of now [10]. Thus, it is imperative to develop computational approaches to predict genome-wide genetic interactions and help complement and enhance wet-lab studies.

**Figure 1:**
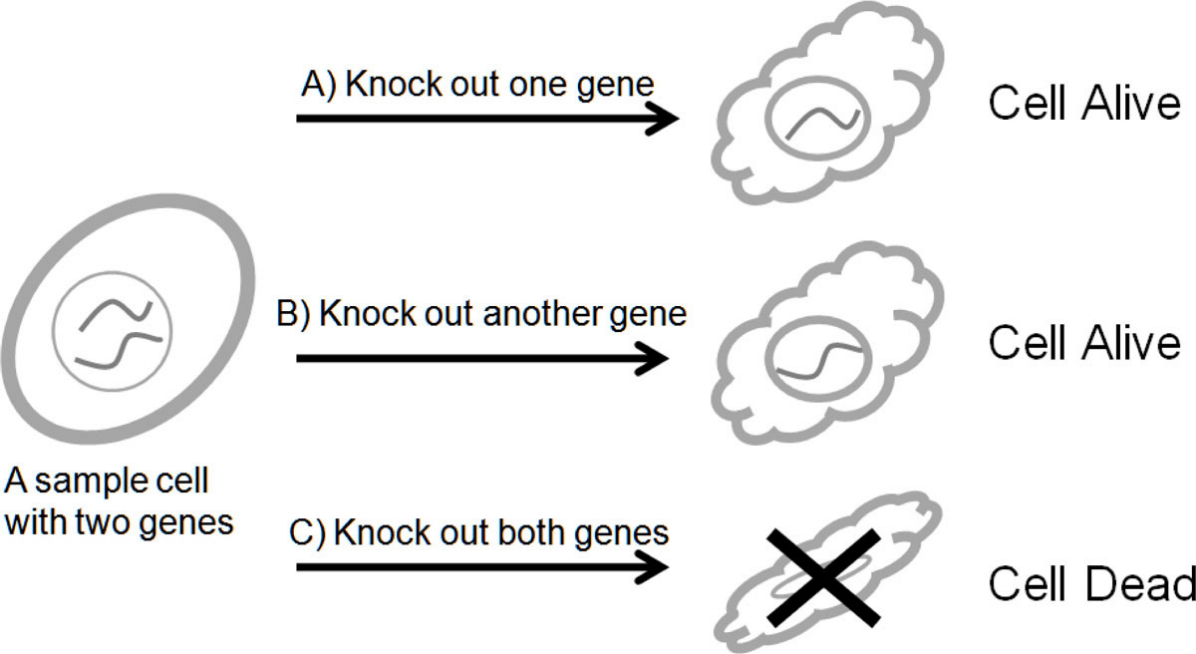
**Illustration of synthetic lethal genetic interaction**. A) and B) the cell is still alive after knocking out one gene; C) the cell died after knocking out both genes.

In extreme cases, mutation of two nonessential genes can lead to lethal phenotype; this kind of genetic interaction is called synthetic lethal genetic interaction (SLGI). Figure 1 illustrates one such synthetic lethal genetic interaction. The SLGIs are of interest because they are able to reveal functional relationships between proteins, pathways and complexes [11-13]. Two synthetic lethal genes have high probability of occurrence in compensatory pathways [14] or compensatory complexes [15]. Furthermore, the SLGIs are potentially useful in finding drug targets or drug combinations [16].

Prediction of SLGIs is impeded by the limit of understanding of genetic interactions. Unlike protein-protein interactions that are known as physical dockings among proteins, the molecular mechanism under genetic interactions has not been fully understood. Thus, it is difficult to select features and understand how features are related to SLGIs. Several computational approaches have been proposed for prediction of SLGIs, and many features, such as protein interactions, gene expression, functional annotation, gene location, protein network characteristics, and genetic phenotype, have been used by these approaches [10,17-20]. However, those methods depend on other genome-wide experimental results.

It is known as a virtual axiom in biology that the "sequence specifies structure and structure determines functionality" [21]. We hypothesize that it is possible to predict the SLGIs using the characteristics of protein sequence alone. Recently, we demonstrated that the yeast synthetic lethal genetic interactions can be explained by the genetic interactions between domains of those proteins [22]. Representing the structures and function of proteins, protein domains are usually regarded as building blocks of proteins and are conserved during evolution. Our studies showed that the domain genetic interactions are new type of relationship between protein domains. Moreover, we found that different domains in multi-domain yeast proteins contribute to their genetic interactions differently. The domain genetic interactions help define more precisely the function related to the synthetic lethal genetic interactions, and then help understand how domains contribute to different functionalities of multi-domain proteins. Using the probabilities of domain genetic interactions, we were able to predict novel yeast synthetic lethal genetic interactions.

However, the feasibility of domain based prediction is limited by the coverage of protein domains. For example, only 4480 of more than 6700 yeast proteins contain PfamA domains. In this study, we used the short polypeptide sequences, which are typically shorter than the classically defined protein domains, to characterize the functionalities of proteins. We demonstrated that the genetic interaction between a pair of proteins can be determined by the genetic interactions between the short polypeptide clusters of those proteins. Using short polypeptide clusters as features, we can not only increase the prediction coverage, but also improve the prediction performance.

## Results

### Identifying significant short polypeptide sequence pairs

**Figure 2:**
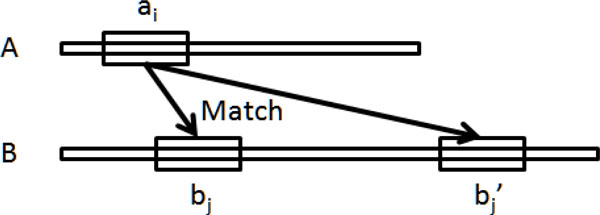
**Illustration of matching between significant short polypeptide sequence pairs**.

We constructed the short polypeptide clusters in three steps. First, we identified significant short polypeptide sequence pairs from yeast proteins based on the similarities of local alignments. For each yeast protein *A*, we chopped its protein sequence into short polypeptides with length of *L *sequentially in a moving window size *w*. Then, we used the Smith-Waterman algorithm [23] to search all local matches with similarity scores beyond a predefined threshold for each short polypeptide of protein *A *against the sequences of all other yeast proteins (Figure 2). We used BLOSUM62 to score the similarity. A significant local match between a short polypeptide sequence *ai *from protein *A *and a short polypeptide sequence *bj *from protein *B *indicates a polypeptide sequence pair *ai *and *bj*. In addition, if using *bj *from protein *B *as query can find *ai *from protein *A *as a significant local match, the short polypeptide pair *ai *and *bj *is identified as a *significant polypeptide sequence pair*.

The significance values of local matching of short polypeptide sequences are determined using a p-value threshold. For each short polypeptide, We compared each short polypeptide to all other short polypeptide sequences and obtained a series of similarity scores. We then modeled those scores using an extreme value distribution. Based on a predefined p-value threshold, we determined the similarity score threshold for local matching. A local match is significant if its score beyond the similarity score threshold. For each short polypeptide sequence, with the same p-value, the similarity score threshold to determine the significant local matching are different.

We considered following parameters to experiment with our method: 1) size L of the short polypeptide sequence; 2) moving window size *w*; 3) penalty for gap and mismatch in the alignment; 4) p-value for the threshold of similarity score. In this study, we chose the size of each short polypeptide sequence *L *to be 25 and the window *w *was set to 5. The penalty for gap and mismatch was chosen to be 14. And, we have used p-value equal to 10^−6 ^for the threshold of similarities. We eventually obtained 3,353,962 short polypeptide sequence pairs covering 6711 yeast proteins. And there are totally 357,256 unique polypeptide sequences involved in these polypeptide sequence pairs.

### Clustering short polypeptide sequences

After identifying significant polypeptide sequence pairs, we developed a clustering algorithm to group similar short polypeptide sequences into clusters. Initially, each significant polypeptide sequence pair was considered as a polypeptide sequence cluster. We first align the significant polypeptide sequence pair using ClustalW [24]. Then, we built a hidden Markov model (HMM) using the output of the multiple sequence alignment as the seed. The HMM model is constructed by the HMMbuild tool from HMMER [25]. After that, we searched the similar short polypeptide sequences using HMM model against all 357,256 short polypeptide sequences. The HMMsearch in HMMER [25] was used to screen similar polypeptide sequences with significances beyond a threshold. The similar short polypeptide sequences were added to the cluster. Then, the above process was repeated until no new short polypeptide sequence was added.

Several stringent thresholds (10^−10^, 10^−15 ^and 10^−20^) for HMMsearch were tested in order to include all similar short polypeptide sequences and reduce false cluster members. Due to the large size of the short polypeptide sequence pairs, we first obtained a cluster using each short polypeptide sequence pair as the seed. Then, we post-processed the short polypeptide sequence clusters. This strategy allowed us to easily run the clustering algorithm on a computer cluster.

### Post-processing short polypeptide clusters

**Figure 3:**
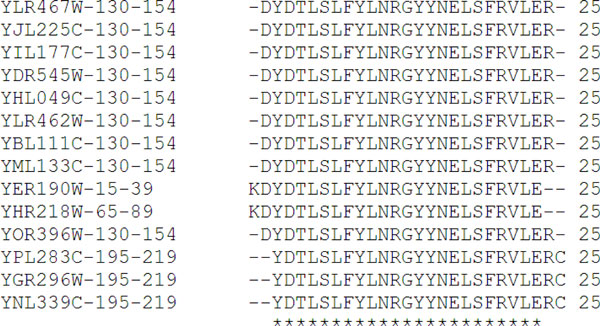
**Multiple sequence alignment of a merged short polypeptide cluster**. The stars in the bottom of the figure indicate the conserved identical amino acids.

Our goal is to use the short polypeptide clusters to represent the functionalities of proteins, like the protein domains were used in [26]. First, we removed the duplicate short polypeptide clusters. Second, we merged two short polypeptide clusters together under 3 conditions: if the clusters share: 1) one polypeptide sequence; 2) 10% of polypeptide sequences of smaller cluster; and 3) 20% of polypeptide sequences of smaller cluster. Although we used a loose merging criterion, the stringent thresholds used by HMMsearch still allowed the short polypeptides in each cluster to have high similarity. Figure 3 shows the multiple sequence alignment of short polypeptides in a merged cluster with 14 short polypeptides. We observed that those short polypeptide sequences in this cluster are highly conserved with significant number of identical amino acids.

Then, we filtered out the short polypeptide clusters with only two polypeptide sequences as those cluster will have no power to predict the SLGIs. We also filtered out short polypeptide clusters that existed in a large number of proteins. Those common short polypeptide clusters exist in both positive and negative data and also do not provide prediction power.

Next, we investigated how the choice of thresholds for HMMsearch and for filtering clusters affects the short polypeptide clusters. We tested different threshold configurations that combined one of three E-value thresholds for HMMsearch: 10^−10^, 10^−15^, 10^−20^; and one of four thresholds for filtering out short polypeptide clusters: 20, 50, 100, and No Filter. Table 1, 2 and 3 list the number of retrained short polypeptide clusters, the number of proteins and the number of SLGIs covered by the short polypeptide clusters, respectively. In three tables, the first column lists the thresholds for filtering out short polypeptide clusters; the first row lists the threshold for cluster merge and the second row lists the E-value thresholds used by HMMsearch. The results showed that increasing the cluster merge threshold will increase the number of covered proteins and number of short polypeptide clusters. Meanwhile, reducing the E-value threshold from 10^−10 ^to 10^−15 ^also increases the number of covered proteins and number of short polypeptide clusters.

**Table 1:** The number of short polypeptide clusters obtained with various thresholds for HMMsearch, cluster filtering and cluster merge.

Threshold	Merge with at least one overlap			Merge with at least 10% overlap			Merge with at least 20% overlap		
	10^−10^	10^−15^	10^−20^	10^−10^	10^−15^	10^−20^	10^−10^	10^−15^	10^−20^
**20**	4188	5324	5326	4443	5807	5797	4760	6443	6399
**50**	4188	5330	5332	4471	5839	5822	4793	6475	6427
**100**	4188	5330	5332	4479	5848	5831	4803	6485	6432
**No Filter**	4189	5331	5333	4486	5856	5840	4812	6494	6440

**Table 2:** The number of proteins covered with various thresholds for HMMsearch, cluster filtering, and cluster merge.

Threshold	Merge with at least one overlap			Merge with at least 10% overlap			Merge with at least 20% overlap		
	10^−10^	10^−15^	10^−20^	10^−10^	10^−15^	10^−20^	10^−10^	10^−15^	10^−20^
**20**	4262	4556	4561	4661	4911	4907	4840	5063	5061
**50**	4262	4585	4590	4858	5073	5058	5050	5201	5216
**100**	4262	4585	4590	4947	5176	5135	5150	5303	5253
**No Filter**	5693	5630	5630	5693	5630	5630	5693	5630	5630

**Table 3:** The number of SLGIs retained with various thresholds for HMMsearch, cluster filtering, and cluster merge.

Threshold	Merge with at least one overlap			Merge with at least 10% overlap			Merge with at least 20% overlap		
	10^−10^	10^−15^	10^−20^	10^−10^	10^−15^	10^−20^	10^−10^	10^−15^	10^−20^
**20**	6143	6648	6650	7110	7386	7408	7289	7820	7873
**50**	6143	6800	6802	7604	7681	7757	7770	8109	8370
**100**	6143	6800	6802	7710	7923	7951	8064	8376	8522
**No Filter**	9592	9427	9427	9592	9427	9427	9592	9427	9427

However, further reducing the E-value threshold to 10^−20 ^did not change the number of covered proteins and number of short polypeptide clusters.

The results showed that the short polypeptide clusters covered more proteins. For example, 5073 proteins are covered by the short polypeptide clusters obtained using 10^−15 ^as HMMsearch threshold, 10% as cluster merge threshold and 50 as polypeptide cluster filtering threshold, comparing to 4480 proteins covered by PfamA domains. The maximum number of polypeptide clusters contained by a protein is 54 using this parameter configuration. The results indicate that the coverage of short polypeptide clusters in yeast proteins is higher than that using PfamA domains. The results also showed that the short polypeptide clusters covered similar number of SLGIs. For example, compared to 7702 SLGIs covered by PfamA domains, our polypeptide clusters cover 7681 SLGIs.

### Predicting yeast synthetic lethal genetic interactions using short polypeptide clusters by maximum likelihood estimation (MLE) approach

In order to demonstrate the superiority of using short polypeptide clusters to predict SLGIs, we first obtained the probabilities of genetic interactions between short polypeptide clusters, and then used them to predict the probabilities of yeast SLGIs. We assumed that the genetic interaction between two short polypeptide clusters is independent and applied the Maximum Likelihood estimation (MLE) approach to estimate the probabilities of short polypeptide clusters.

We compared the MLE methods based on short polypeptide clusters, obtained using different HMMsearch, cluster filtering and cluster merge thresholds, to the MLE method based on protein domains. We trained those MLE methods using all SLGIs covered by features. The MLE based on short polypeptide clusters were able to assign the probabilities of genetic interaction to more protein pairs. For example, the MLE method based on short polypeptide clusters using 10^−15 ^as HMMsearch threshold, 10% as cluster merge threshold and 50 as polypeptide cluster filtering threshold were able to assign the probabilities of genetic interaction to 1,060,860 protein pairs while the MLE based protein domains can only assign probabilities of genetic interaction to 536,175 protein pairs. This result showed that short polypeptide cluster based approach provides a much higher coverage to predict SLGIs.

To further evaluate the performance of short polypeptide cluster based MLE method, we tested the MLE methods on an experimentally obtained genetic interactions and non-genetic interactions, which include 3771 SLGIs and 688,045 non- SLGIs [10]. This data have been used by Wong et al. [10]. So we refer this experimental data as Wong data. We first trained the MLE method using SLGIs that are not included in the Wong data. Then, we assigned the probabilities of genetic interaction to the SLGIs and non SLGIs in Wong data. Table 4 lists the AUC (area under ROC curve) values for predicting Wong data of MLE methods based on short polypeptide clusters obtained using different HMMsearch, cluster merge and cluster filtering thresholds. The performance of MLE based on short polypeptide clusters is slightly better than that of MLE method based on protein domains. The AUC score for MLE based on short polypeptide clusters using 10^−15 ^as HMMsearch threshold, 10% as cluster merge threshold and 50 as polypeptide cluster filtering threshold is 0.6761 while the AUC score for MLE based on protein domains is 0.6567.

**Table 4:** The AUC values for the predictions of Wong data using different short polypeptide clusters obtained with various thresholds for HMMsearch cluster filters, and cluster merges.

Threshold	Merge with at least one overlap			Merge with at least 10% overlap			Merge with at least 20% overlap		
	10^−10^	10^−15^	10^−20^	10^−10^	10^−15^	10^−20^	10^−10^	10^−15^	10^−20^
**20**	0.630	0.639	0.636	0.644	0.647	0.642	0.627	0.665	0.627
**50**	0.630	0.638	0.634	0.669	**0.676**	0.644	0.662	0.671	0.633
**100**	0.630	0.659	0.634	0.644	0.628	0.645	0.624	0.628	0.636

## Discussion and conclusions

In this study, we developed a framework to identify significant short polypeptide clusters from yeast protein sequences. We hypothesized that those short polypeptide clusters represent the functionalities of proteins, like the protein domains. We then used these short polypeptide clusters as features to predict yeast synthetic lethal genetic interactions. The short polypeptide cluster based approach provides much higher coverage for predicting yeast synthetic lethal genetic interactions. Evaluation using experimental data sets showed that the short polypeptide cluster based approach can achieve higher performance than the previous protein domain based approach.

In future, we would like to continue improve the identification of short polypeptide clusters. Moreover, it is worthwhile to develop methods to understanding those short polypeptide clusters. Annotating those short polypeptide clusters may help better understand the functionalities of protein domains.

## Methods

### Source of data

We downloaded the yeast synthetic lethal genetic interactions from the *Saccharomyces *Genome Database (SGD) [27] (February 2011 version). There were totally 11011 synthetic lethal genetic interactions. We downloaded the protein sequences of yeast from GenBank [28]. There are totally 6717 proteins with sequences. The minimum and maximum lengths of the protein sequences in yeast are 16 and 4901 respectively. The average length is 450 and the standard deviation of the protein sequences is 380.

### Determination of local alignment similarity score threshold

The distribution of scores of local alignments between a short polypeptide and all other short polypeptide sequences can be described by extreme value distribution (EVD):

(1) $$F\left(x\right)=e^{-e^{\frac{x-\mu }{\beta }}}$$

and the parameters of the EVD can be estimated by:

(2) $$\beta =\frac{\sigma \sqrt{6}}{\pi }$$

(3) $$\mu =\bar{X}-0.5772\beta$$

where $\bar{X}$and σ are the sample mean and standard deviation, respectively.

Based on Karlin-Altshcul statistics [29], the expected number of high-scoring segment pairs (HSPs) with score higher than S can be obtained by:

(4) $$E=Kmne^{-\lambda S}$$

where *m *and *n *are the lengths of the two sequences being compared. The parameter K and λ can be obtained from parameters of the EVD:

(5) λ=1/β

(6) $$K=e^{\frac{\mu }{\beta }}/mn$$

The p-value of finding at least one HSP with score higher than S can be obtained by [29]:

(7) $$P=1-e^{-E}$$

With a given p-value, we can get a corresponding E-value. The parameter *K *and λ can be estimated by sample mean and standard deviation of scores. Significant similarity score can be computed by equation (4).

### Algorithm to cluster short polypeptide sequences

The short polypeptide sequence clustering method implemented is summarized as follows:

Input: a pair of short polypeptide sequences

Initialization: add the short polypeptide sequence pair into cluster

Step 1. Conduct multiple sequence alignment (MSA) for the sequences in the cluster using ClustalW;

Step 2. Build a HMM model using HMMbuild from the output of MSA in step 1;

Step 3. Search all similar short polypeptide sequences using HMMsearch and add them to the cluster. If no new short polypeptide sequence is added, stop. Else, go back to step 1.

### Estimation of probabilities and significances of domain genetic interactions

We treated the protein SLGIs *L_m,n _*and short polypeptide cluster genetic interactions *C_i,j _*as random variables. *L_m,n_*=1 if two proteins *i *and *j *genetically interact and *L_m,n _*=0 otherwise. *C_i,j_*=1 if two short polypeptide clusters *i *and *j *genetically interact and *C_i,j_*=0 otherwise. We estimated the probabilities of potential short polypeptide cluster interactions Pr(*C_i,j_*=1) by maximizing the likelihood of observed genetic interactions using the Expectation-Maximization (EM) algorithm [30-32]. The EM algorithm iteratively estimates the maximum likelihood of the 'complete data' that combine the observed data and unobserved data. Here, the protein genetic interactions and the short polypeptide cluster information of proteins are our observed data and the short polypeptide cluster genetic interactions are our unobserved data.

Assuming short polypeptide cluster genetic interactions are independent, the likelihood of observed protein genetic interactions based on short polypeptide cluster genetic interactions can be obtained as:

(8) $$L=\underset{i,j}{\prod }\text{Pr}{\left(C_{i_{,}j}=1\right)}^{M_{i,j}+a}{\left(1-\text{Pr}\left(C_{i_{,}j}=1\right)\right)}^{N_{i,j}+K_{i,j}+b}$$

where M_i,j _is the number of genetic interacting pairs between short polypeptide clusters i and j in all protein genetic interactions; N_i,j _is the number of non genetic interacting short polypeptide cluster pairs between i and j in protein genetic interactions; and K_i,j _is the number of non genetic interacting protein pairs including i in one protein and j in the other one. The value of K_i,j _is computed by counting all possible protein pairs with i in one protein and j in the other one with excluding the known genetic interacting protein pairs. The K_i,j _will remain unchanged during EM computation. The constants *a *and *b *are pseudo counts to avoid the Pr(C_i,j _=1) or Pr(C_i,j _=0) to be zero when instances of domains i and j are rare. We set both *a *and *b *to 1 in our calculation.

Initially, M_i,j _was set to the number of genetic interactions between domain i and j in experimental genetic interactions; N_i,j _is set to 0. And Pr(C_i,j _=1) was initialized as following:

(9) $$\text{Pr}\left({\text{C}}_{\text{i,j}}=1\right)=\frac{{\text{M}}_{\text{i,j}}}{{\text{M}}_{\text{i,j}}+N_{i,j}+K_{i,j}}$$

In each Expectation step of EM algorithm, we first estimated the expected values of E[M_i,j_] and E[N_i,j_] [31] using the current Pr(C_i,j _=1):

(10) $$E\left[M_{i,j}\right]=\underset{m,n}{\sum }\left[\frac{\text{Pr}\left(C_{i,j}=1\right)}{1-\Pi_{i\subset C\left(m\right),j\subset C\left(n\right)}\left(1-\text{Pr}\left(C_{i,j}=1\right)\right)}\right]$$

(11) $$E\left[N_{i,j}\right]=\underset{m,n}{\sum }\left(1-\left[\frac{\text{Pr}\left(C_{i,j}=1\right)}{1-\Pi_{i\subset C\left(m\right),j\subset C\left(n\right)}\left(1-\text{Pr}\left(C_{i,j}=1\right)\right)}\right]\right)$$

Then, we calculate the Pr(C_i,j _=1) using the E[M_i,j_] and E[N_i,j_] as following (Maximization step):

(12) $$\text{Pr}\left(C_{i,j}=1\right)=\frac{E\left[M_{i,j}\right]+a}{E\left[M_{i,j}\right]+E\left[N_{i,j}\right]+K_{i,j}+a+b}$$

The EM algorithm was over the Expectation and Maximization steps until the change of likelihood L is less than a pre-defined small value.

We assumed that two proteins genetically interact (*L_m,n _*=1) if and only if at least one domain pair from the two proteins genetically interact (*C_i,j _*_=1_). Then, we calculated the probability of two proteins genetically interacting Pr(*L_m,n _*=1) as following:

(13) $$\text{Pr}\left(L_{m,n}=1\right)=1.0-\underset{\begin{array}{c} i\in C\left(m\right)\\ j\in C\left(n\right) \end{array}}{\prod }\left(1-\text{Pr}\left(C_{i,j}=1\right)\right)$$

A pair of proteins was predicted to be SLGI only if its probability is higher than a predefined threshold.

## Competing interests

The authors declare that they have no competing interests.

## Authors' contributions

FL and PKS designed research. BL and YHZ implemented the idea. FL, YHZ and XWC analyzed the results. All authors have read and approved the final manuscript.
